# A diminished sciatic nerve structural integrity is associated with distinct peripheral sensory phenotypes in individuals with type 2 diabetes

**DOI:** 10.1007/s00125-023-06050-y

**Published:** 2023-11-29

**Authors:** Christoph M. Mooshage, Dimitrios Tsilingiris, Lukas Schimpfle, Lukas Seebauer, Omar Eldesouky, Taraneh Aziz-Safaie, Anja Hohmann, Stephan Herzig, Julia Szendroedi, Peter Nawroth, Sabine Heiland, Martin Bendszus, Felix T. Kurz, Stefan Kopf, Johann M. E. Jende, Zoltan Kender

**Affiliations:** 1 Department of Neuroradiology, Heidelberg University Hospital, Heidelberg, Germany; 2 Department of Endocrinology, Diabetology, Metabolism and Clinical Chemistry (Internal Medicine 1), Heidelberg University Hospital, Heidelberg, Germany; 3 German Center for Diabetes Research (DZD), München-Neuherberg, Germany; 4 First Department of Internal Medicine, University Hospital of Alexandroupolis, Democritus University of Thrace, Alexandroupolis, Greece; 5 Department of Neurology, Heidelberg University Hospital, Heidelberg, Germany; 6 Institute for Diabetes and Cancer (IDC), Helmholtz Diabetes Center, Helmholtz Center, Munich, Neuherberg, Germany; 7 Joint Heidelberg-IDC Translational Diabetes Program, Inner Medicine 1, Heidelberg University Hospital, Heidelberg, Germany; 8 Division of Experimental Radiology, Department of Neuroradiology, Heidelberg University Hospital, Heidelberg, Germany; 9 German Cancer Research Center, Heidelberg, Germany

**Keywords:** Diabetic neuropathy, Diffusion tensor imaging, Fractional anisotropy, Magnetic resonance neurography, Quantitative sensory testing, Sensory profiling, Type 2 diabetes

## Abstract

**Aims/hypothesis:**

Quantitative sensory testing (QST) allows the identification of individuals with rapid progression of diabetic sensorimotor polyneuropathy (DSPN) based on certain sensory phenotypes. Hence, the aim of this study was to investigate the relationship of these phenotypes with the structural integrity of the sciatic nerve among individuals with type 2 diabetes.

**Methods:**

Seventy-six individuals with type 2 diabetes took part in this cross-sectional study and underwent QST of the right foot and high-resolution magnetic resonance neurography including diffusion tensor imaging of the right distal sciatic nerve to determine the sciatic nerve fractional anisotropy (FA) and cross-sectional area (CSA), both of which serve as markers of structural integrity of peripheral nerves. Participants were then assigned to four sensory phenotypes (participants with type 2 diabetes and healthy sensory profile [HSP], thermal hyperalgesia [TH], mechanical hyperalgesia [MH], sensory loss [SL]) by a standardised sorting algorithm based on QST.

**Results:**

Objective neurological deficits showed a gradual increase across HSP, TH, MH and SL groups, being higher in MH compared with HSP and in SL compared with HSP and TH. The number of participants categorised as HSP, TH, MH and SL was 16, 24, 17 and 19, respectively. There was a gradual decrease of the sciatic nerve’s FA (HSP 0.444, TH 0.437, MH 0.395, SL 0.382; *p*=0.005) and increase of CSA (HSP 21.7, TH 21.5, MH 25.9, SL 25.8 mm^2^; *p*=0.011) across the four phenotypes. Further, MH and SL were associated with a lower sciatic FA (MH unstandardised regression coefficient [B]=−0.048 [95% CI −0.091, −0.006], *p*=0.027; SL B=−0.062 [95% CI −0.103, −0.020], *p*=0.004) and CSA (MH β=4.3 [95% CI 0.5, 8.0], *p*=0.028; SL B=4.0 [95% CI 0.4, 7.7], *p*=0.032) in a multivariable regression analysis. The sciatic FA correlated negatively with the sciatic CSA (*r*=−0.35, *p*=0.002) and markers of microvascular damage (high-sensitivity troponin T, urine albumin/creatinine ratio).

**Conclusions/interpretation:**

The most severe sensory phenotypes of DSPN (MH and SL) showed diminishing sciatic nerve structural integrity indexed by lower FA, likely representing progressive axonal loss, as well as increasing CSA of the sciatic nerve, which cannot be detected in individuals with TH. Individuals with type 2 diabetes may experience a predefined cascade of nerve fibre damage in the course of the disease, from healthy to TH, to MH and finally SL, while structural changes in the proximal nerve seem to precede the sensory loss of peripheral nerves and indicate potential targets for the prevention of end-stage DSPN.

**Trial registration:**

ClinicalTrials.gov NCT03022721

**Graphical Abstract:**

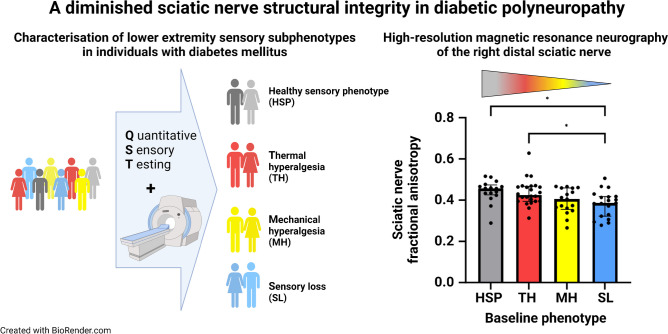

**Supplementary Information:**

The online version contains peer-reviewed but unedited supplementary material available at 10.1007/s00125-023-06050-y.


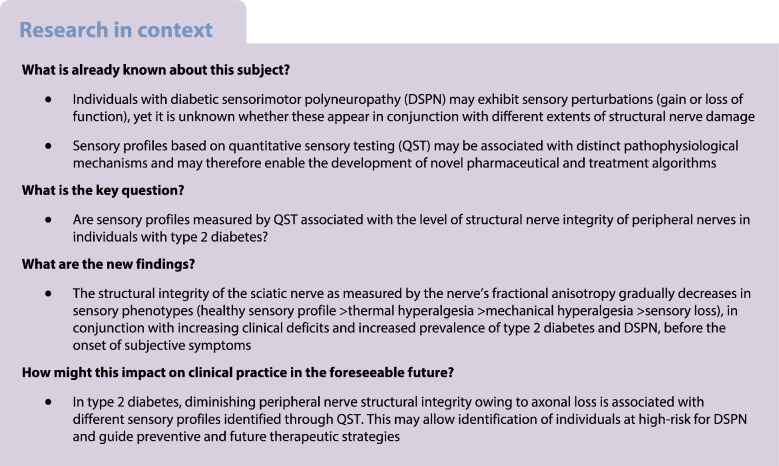


## Introduction

Diabetic sensorimotor polyneuropathy (DSPN) embodies the most common type of neuropathy in individuals with diabetes, affecting up to half of those with type 2 diabetes [1]. Painful subtypes of DSPN are a major reason for the impairment of quality of life caused by the disease, with a substantial economic burden due to the resultant healthcare costs [2, 3]. However, to date, the pathophysiology and the natural course of the disease are incompletely understood and therapies are limited to symptomatic strategies [4, 5]. Novel diagnostic approaches now aim to identify distinct sensory phenotypes in individuals with diabetes, with the goal of improving patient care by identifying those at risk and enabling individual prognoses on the progression of DSPN, thereby potentially contributing to the elucidation of the natural course and pathophysiological of the disease [6–8]. Quantitative sensory testing (QST), a neurophysiological and psychological test battery of 13 sensory variables introduced by the German Research Network on Neuropathic Pain (DFNS) to assess large and small-fibre functions [9], has recently been applied to stratify individuals with polyneuropathy into four sensory clusters: healthy; sensory loss (SL); thermal hyperalgesia (TH); and mechanical hyperalgesia (MH) [7]. SL is characterised by a loss of small- and large-fibre function with coexisting paradoxical heat sensations (PHS). In TH the function of large and small fibres is relatively preserved, with the key feature being cold and heat hyperalgesia. Individuals in the MH cluster suffer from diminished cold and heat sensory functions, while pressure and pinprick hyperalgesia, as well as dynamic mechanical allodynia (DMA), are present. While these sensory clusters were suggested to be related to distinct pathophysiological mechanisms [7], it remains unclear whether they define entirely different phenotypes or whether they reflect an order of severity and chronological sequence of nerve fibre damage in individuals with polyneuropathies such as DSPN. It could, however, be hypothesised, that from a functional standpoint, the MH and SL clusters exhibiting loss of thermal sensation and loss of both thermal and mechanical sensation, respectively, may represent the most severe phenotypes in DSPN.

High-resolution magnetic resonance neurography (MRN) at 3 Tesla (3T) represents an additional sophisticated method that has provided valuable insights into pathophysiological mechanisms of DSPN in individuals with type 2 diabetes through the application of multimodal sequence techniques [10–12]. Diffusion tensor imaging (DTI), a technique that measures the restricted diffusion of water molecules along the axons of peripheral nerves, allows analysis of the structural integrity of peripheral nerves in vivo [13]. The fractional anisotropy (FA) embodies the main readout parameter showing correlations with electrophysiological variables of axonal and myelin sheath integrity and thereby reflects the structural integrity of peripheral nerves [14–16].

Consequently, DTI-MRN and sensory profiling by QST were combined to investigate whether the structural integrity of the sciatic nerve differs between the four clusters of sensory phenotypes and to test the hypothesis of whether nerve damage may follow a predefined cascade in individuals with type 2 diabetes.

## Methods

### Study design and participants

This cross-sectional study was approved by the local ethics committee of Heidelberg University Hospital (Heidelberg Study on Diabetes and Complications [HEIST-DiC]; ClinicalTrials.gov registration no. NCT03022721; local ethics no. S-383/2016) and was conducted according to the principles of the declaration of Helsinki. All participants gave written informed consent. Sex was investigator assessed or self-reported. The study took place between January 2016 and November 2019. Out of a total of 262 participants with type 2 diabetes in the HEIST-DiC study, 76 were included in the current study, all 76 having undergone MRN of the right leg (22 women, 54 men) and had a full set of MRN, QST, electrophysiological, clinical and laboratory data. A cohort of individuals without type 2 diabetes, which did not differ from the cohort of individuals with type 2 diabetes regarding age, sex and BMI and having undergone identical phenotyping were examined as a control group (*n*=13). The screening and recruitment of the participants, as well as QST, electrophysiological and serological examinations of all participants, were performed by the department of Endocrinology, Diabetology and Clinical Chemistry (Internal Medicine 1) [15]. Sex and gender were not considered during the process of screening and recruitment of participants.

To preclude factors other than type 2 diabetes as being responsible for peripheral nerve damage we excluded individuals suffering from other conditions predisposing to peripheral neuropathy (alcoholism, hypovitaminosis, malignant or infectious diseases, monoclonal gammopathy, chronical neurological diseases such as multiple sclerosis, or previous exposure to neurotoxic agents), as described previously [15]. Further, we excluded individuals with a history of lumbar surgery or disk protrusion, those with contraindications for MRN, pregnant women and individuals under 18 years of age or with an eGFR <60 ml/min per 1.73 m^2^[15]. Subsequently, 239 individuals with type 2 diabetes were eligible to participate in this study. Of these, 76 underwent the full study protocol consisting of DTI-MRN, QST of the right foot and electrophysiological as well as serological exams and were subsequently included in cross-sectional analysis. To justify the appropriateness of the size of the cohort, we performed a post hoc power analysis based on a previously published study investigating associations of DTI-MRN parameters with clinical, serological and electrophysiological variables [15], which yielded a power of 0.83. Subsequently for an α level of 0.05, an effect size of 0.4 and a power of 0.8, 76 participants were required to conduct perform one-way ANOVA for four groups.

### Clinical and electrophysiological examination

Blood was drawn and urine was taken under fasting conditions in the morning, and samples were immediately processed in the Central Laboratory of University Hospital of Heidelberg under standardised conditions. HbA_1c_, lipid profile, urinary albumin/creatinine ratio (ACR), eGFR, high-sensitivity troponin T (hsTNT) and high-sensitivity C-reactive protein (hsCRP) were assessed. The eGFR was calculated using the Chronic Kidney Disease Epidemiology Collaboration (CKD-EPI) formula [17] and ACR was determined in mg/g.

To evaluate the presence of neuropathic symptoms, the neuropathy symptom score (NSS) was estimated, while clinical neurological deficits of the lower extremities were evaluated using the neuropathy disability score (NDS). Nerve conduction studies on each participant’s right leg included measurements of nerve conduction velocity (NCV) and sensory nerve action potential (SNAP) of the sural nerve. NCVs as well as compound motor action potentials (CMAP) of the common peroneal and tibial nerves were performed maintaining a skin temperature of at least 32°C. Non-detectable excitation of the sural nerve bilaterally was considered indicative of advanced sural nerve damage. The corresponding missing NCV and SNAP values were imputed with the lowest values measured in the HEIST-DiC cohort (30.9 m/s and 2.54 μV, respectively). As additional sensitivity analysis for group comparisons, sural NCV and SNAP as well as common peroneal and tibial NCVs and CMAPs were converted into categorical variables (normal/abnormal) using the 2.5 percentile values in the HEIST-DiC cohort (38.6, 37.0, 38.0 m/s and 2.54, 1.14, 2.72 μV for sural, common peroneal and tibial nerves, respectively) as cut-offs.

### QST and diagnosis of neuropathy

**Fig. 1:**
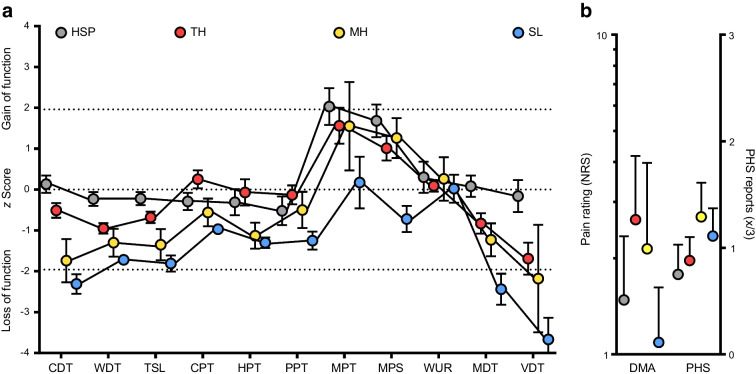
Graphic presentation of the 13-domain QST profiles of the four subgroups of participants with type 2 diabetes. (**a**) Presentation of the 11 *z*-transformed QST domains. The *z*-transformation normalises differences due to age, sex and tested anatomical region. Positive and negative *z* scores denote gain (hyperalgesia) or loss (hypoalgesia or hypoaesthesia) of function, respectively. (**b**) Presentation of numeric pain ratings for dynamic mechanical allodynia (0–100) on a log_10_ scale and of frequency of paradoxical heat sensation (×/3 denotes number of paradoxical heat sensations [0–3] for 3× repeated testing). Grey circles, HSP, (*n*=16); red circles, TH (*n*=24); yellow circles, MH (*n*=17); blue circles, SL (*n*=19). MPT, mechanical pain threshold; NRS, numerical rating scale; PPT pressure pain threshold; WUR, wind-up ratio

Participants underwent QST according to the protocol of the DFNS as described in detail elsewhere [11, 18]. The 13 domains of the DFNS QST protocol (cold detection threshold, warm detection threshold, perception of alternating warm and cold stimuli including PHS, cold and heat pain thresholds, mechanical and vibration detection thresholds, pinprick and blunt pressure pain thresholds, stimulus/response functions for pinprick sensitivity, DMA and pain summation to repetitive pinprick stimuli, known as the wind-up ratio) were measured on the right foot. Participants were subsequently grouped in to the four outlined sensory phenotypes according to the deterministic version of the sorting algorithm established by Vollert et al [8]. Using this algorithm, based on the greatest concordance of the 13 items of the QST profile, each participant with type 2 diabetes was assigned to a single one of the four sensory phenotypes (Fig. 1): (1) healthy sensory profile (HSP), representing individuals with type 2 diabetes whose sensory profile resembled those of healthy individuals; (2) TH; (3) MH; and (4) SL. [8]

According to current guidelines, the diagnosis of DSPN through the sole performance of QST is not recommended, so we applied the Toronto consensus criteria to diagnose ‘confirmed neuropathy’[19] in order to investigate whether sensory profiles are associated with established diagnostic criteria of DSPN. Diagnosis of DSPN was given where there were abnormal results of the nerve conduction study of the sural nerve and additional abnormal nerve conduction studies of the common peroneal or tibial nerve together with an NSS and/or NDS of ≥3.

### MRI imaging protocol and data analysis

#### Magnetic resonance neurography imaging protocol

High-resolution MRN of the right leg was performed in a 3.0 Tesla MR scanner (Magnetom TIM-TRIO; Siemens, Erlangen, Germany) using a 15-channel transmit–receive extremity coil with the participant positioned in the supine position. An axial high-resolution T2-weighted (T2w) turbo spin echo two-dimensional sequence with spectral fat saturation of the right mid-thigh (A) and a DTI with an axial fat-suppressed, diffusion-weighted two-dimensional echo-planar sequence (B) were applied with the following parameters:

(A) repetition time (TR) 5970 ms, echo time (TE) 55 ms, field of view (FOV) 160×160 mm^2^, matrix size 512×512, slice thickness 4 mm, interslice gap 0.35 mm, voxel size 0.3×0.3×4.0 mm^3^, three averages, 24 images.
(B) TR 5100 ms; TE 92.8 ms; b=0 and 1000 s/mm^2^; directions 20; FOV 160×160 mm^2^; matrix size 128×128; slice thickness 4 mm; voxel size 1.3×1.3×4 mm^3^; no interslice gap, three averages, 24 slices, 1512 images.

#### Image post-processing

All images were pseudonymised and post-processed by two neuroradiologists with 3 and 7 years of experience in MRN imaging, respectively. The analysis of DTI was performed using Nordic BRAINEX (NordicNeuroLab, Norway, version 2.2, 2019), an automated software applied for the calculation and reconstruction of fibre tracts in diffusion-weighted imaging. Hereby, the sciatic nerve’s FA was calculated [15, 20]: first, automatic co-registration of the axial T2w sequence with the DTI sequence was performed (B); then the anatomical region of the sciatic nerve was manually delineated and the sciatic nerve’s mean FA was automatically calculated by Nordic BRAINEX. Settings for DTI analysis were chosen as follows and as performed previously: value of >0.1 for the nerve’s FA, a tract turning angle of 41.4°, a minimum fibre length of 20 mm and one seed per voxel as cut-off values for automated fibre tracking [15, 20]. The FA is a dimensionless parameter, with values ranging between 0 and 1 being representative of the structural integrity of peripheral nerves [15, 21, 22] and associated with electrophysiological markers of axonal and myelin sheath integrity [12, 15].

The manual anatomical segmentation of the tibial compartment of the sciatic nerve on the axial fat-saturated turbo spin echo two-dimensional sequence of the right mid-thigh was performed using ImageJ (US National Institutes of Health, Bethesda, Maryland, USA, version 1.53k) and analysed the mean cross-sectional area (CSA).

#### Statistical analysis

All statistical analyses were performed using GraphPad Prism 9 (GraphPad Software, La Jolla, CA, USA) and SPSS 28 (IBM, Armonk, NY, USA). Dichotomous variables are presented as absolute numbers and percentages. The normality of the distribution of continuous variables was tested using the Kolmogorov–Smirnov test. Normally and non-normally distributed quantitative variables are presented as means (SD) or medians (25, 75 IQR). Comparisons across the four phenotype groups (HSP, SL, TH, MH) regarding qualitative characteristics were made with the χ^2^ test; quantitative variables were compared using one-way ANOVA or the Kruskal–Wallis non-parametric test, depending on the normality of their distribution. Where there were significant differences across the four groups, between-group post hoc comparisons with Bonferroni correction were carried out. To examine the correlations between pairs of continuous variables, the Pearson *r* was calculated, while correlations between categorical and continuous variables were examined via binary logistic regression. To ascertain the independence of observed associations we used partial correlation or multivariable linear regression analysis. Independent variables exhibiting a markedly skewed distribution (e.g. duration of diabetes, hsTNT, ACR) were log-transformed prior to correlation analysis. All statistical tests were two-sided and a *p* value of <0.05 was considered statistically significant.

## Results

Upon application of the sorting algorithm within the type 2 diabetes participant cohort, a total of 16, 24, 17 and 19 participants were categorised as HSP, TH, MH and SL, respectively. In Fig. 1 and the electronic supplementary material (ESM Results and ESM Table 1) we provide QST profiles and group comparisons of each subgroup.

Group comparisons across the four clusters showed no differences in age, sex composition, number of active smokers, BMI, waist circumference, duration of type 2 diabetes, type of glucose-lowering medication, total serum cholesterol, eGFR and hsCRP (Table 1).

**Table 1:** Group comparisons of demographic, serological, electrophysiological and MRN variables of the sciatic nerve of study participants with type 2 diabetes

Variable	HSP (*n*=16)	TH (*n*=24)	MH (*n*=17)	SL (*n*=19)	*p* value	Post hoc tests (after Bonferroni correction)	
						*p* value (SL vs HSP)	*p* value (SL vs TH)
Age, years	61.8 (8.7)	64.3 (7.8)	67.5 (7.0)	65.1 (10.7)	0.154		
Female sex, yes, *n* (%)	5 (31.3)	11 (45.8)	3 (17.6)	3 (15.8)	0.110		
Active smoking, yes, *n* (%)	1 (6.3)	2 (8.3)	2 (11.8)	2 (10.5)	0.948		
BMI, kg/m^2^	29.1 (5.1)	30.2 (3.2)	27.8 (3.9)	30.3 (5.0)	0.240		
Waist circumference, cm	103.4 (13.7)	108.2 (8.2)	108.7 (10.3)	110.9 (14.6)	0.756		
Diabetes duration, years	7.5 (3.5, 14.8)	10.0 (5.5, 16.3)	5.0 (0.5, 12.0)	9.0 (5.0, 17.5)	0.195		
HbA_1c_, mmol/mol	53.3 (9.6)	52.0 (10.8)	51.9 (15.0)	56.1 (14.6)	0.191		
HbA_1c_, %	7.0 (0.9)	6.9 (1.0)	6.9 (1.4)	7.3 (1.3)	0.187		
Glucose-lowering therapy, yes, *n* (%)							
Diet only	4 (25.0)	3 (12.5)	7 (41.2)	4 (21.1)	0.235		
Metformin	11 (68.8)	13 (54.2)	6 (35.3)	12 (63.2)	0.183		
Sulfonylurea	1 (6.3)	0 (0.0)	2 (11.8)	1 (5.3)	0.445		
Glinide	0 (0.0)	1 (4.2)	0 (0.0)	1 (5.3)	0.646		
DPP4i	1 (6.3)	2 (8.3)	4 (23.5)	4 (21.1)	0.358		
GLP1RA	1 (6.3)	1 (4.2)	1 (5.9)	0 (0.0)	0.763		
SGLT2i	1 (6.3)	3 (12.5)	1 (5.9)	1 (5.3)	0.789		
TZD	0 (0.0)	0 (0.0)	0 (0.0)	0 (0.0)	-		
Insulin	3 (18.8)	8 (33.3)	2 (11.8)	4 (21.1)	0.789		
Τotal cholesterol, mmol/l	203.1 (49.4)	188.9 (34.7)	188.8 (41.5)	190.4 (47.2)	0.723		
eGFR, ml/min per 1.73 m^2^	92.5 (12.5)	85.1 (16.5)	88.9 (15.4)	89.7 (18.8)	0.337		
hsCRP, mg/l	1.48 (0.44, 3.54)	1.60 (0.67, 3.34)	1.46 (0.47, 6.71)	0.87 (0.51, 2.11)	0.590		
ACR, mg/mmol	0.07 (0.05, 0.26)	0.11 (0.06, 0.14)	0.12 (0.05, 0.47)	0.39 (0.11, 1.66)	0.034*	0.064	0.069
hsTNT, ng/ml	8.5 (5.8, 9.3)	9.0 (8.0, 11.0)	9.0 (9.0, 12.0)	13.5 (6.5, 16.0)^†^	0.050*	0.042*	0.513
Pain, *n*, %	2 (12.5)	3 (12.5)	6 (35.3)	10 (52.6)^†‡^	0.012*		
ΝSS, *n*/10	4.5 (0.0, 7.8)	5.0 (0.0, 6.8)	4.0 (0.0, 6.5)	6.0 (4.0, 7.0)	0.755		
ΝDS, *n*/10	0.5 (0.0, 2.0)	2.0 (1.3, 5.0)	4.0 (2.0, 5.5)*	6.0 (4.0, 9.0)^†††‡^	<0.001***		
Sural NCV, m/s	42.4 (8.6)	41.6 (8.4)	38.4 (8.8)	35.3 (8.7)	0.058		
Sural SNAP, µV	5.3 (3.5)	4.0 (3.2)	3.1 (2.4)	1.5 (1.0)^†††‡^	0.001**	<0.001***	0.020**
Pathological sural NCV (yes, %)	5 (31.3)	8 (33.3)	10 (58.8)	15 (78.9)^†‡^	0.008**	0.005**	0.003**
Pathological sural SNAP, yes, *n* (%)	4 (25.0)	10 (41.7)	8 (47.1)	17 (89.5)^†††‡‡§^	0.001**	<0.001***	0.001**
Common peroneal NCV, m/s	42.2 (3.7)	41.3 (6.6)	38.8 (6.5)	35.6 (5.6)^†‡^	0.006**		
Common peroneal CMAP, mV	7.0 (2.9)	6.1 (3.2)	4.4 (2.8)	2.9 (2.1)^†††‡‡^	<0.001***		
Pathological common peroneal NCV, yes, *n* (%)	0 (0.0)	5 (20.8)	4 (23.5)	15 (78.9)^†††^	<0.001		
Pathological common peroneal CMAP, yes, *n* (%)	0 (0.0)	1 (4.2)	2 (11.8)	5 (26.3)	0.113		
Tibial NCV, m/s	42.8 (4.1)	39.9 (4.9)	38.3 (5.3)	37.0 (7.3)^†^	0.026*		
Tibial CMAP, mV	14.1 (6.7)	11.8 (6.0)	10.0 (6.5)	6.5 (6.2)^††‡^	0.007**		
Pathological tibial NCV, yes, *n* (%)	2 (12.5)	9 (37.5)	6 (35.3)	13 (68.4)^††^	0.012*		
Pathological tibial CMAP, yes, *n* (%)	1 (6.3)	3 (12.5)	2 (11.8)	5 (26.3)	0.401		
Confirmed neuropathy, yes, *n* (%)	1 (6.3)	7 (29.2)	7 (41.2)*	15 (78.9)^†††‡‡§^	<0.001***		
FA	0.444 (0.055)	0.437 (0.063)	0.395 (0.061)	0.382 (0.064)^†‡^	0.005**	0.024*	0.029*
CSA, mm^2^	21.7 (4.6)	21.5 (4.7)	25.9 (5.4)	25.8 (6.8)	0.011*	0.166	0.191

Values are displayed as *n* (%), means (SD) or medians (25, 75 IQR)

Pathological sural NCV, <38.6 m/s; pathological sural SNAP, <2.54 μV; pathological common peroneal NCV, <37.0 m/s, pathological common peroneal CMAP, <1.14 μV; pathological tibial NCV, <38.0 m/s; pathological tibial CMAP, <2.72 μV

*p<0.05, **p<0.01, ***p<0.001 for difference across the four groups in one-way ANOVA; ^†^*p*<0.05, ^††^*p*<0.01, ^†††^*p*<0.001 for difference vs the healthy subgroup in post hoc pairwise comparisons after Bonferroni correction; ^‡^*p*<0.05, ^‡‡^*p*<0.01 for difference vs the TH subgroup in post hoc pairwise comparisons after Bonferroni correction; ^§^*p*<0.05 for difference vs the MH subgroup in post hoc pairwise comparisons after Bonferroni correction. DPP4i, dipeptidyl peptidase 4 inhibitors; GLP1RA, glucagon-like peptide 1 receptor agonists; SGLT2i, sodium–glucose cotransporter 2 inhibitors; TZD, thiazolidinediones

There was a gradual increase in ACR (median [IQR]: HSP 0.07 [0.05, 0.26] mg/mmol; TH 0.11 [0.06, 0.14] mg/mmol; MH 0.12 [0.05, 0.47] mg/mmol; SL 0.39 [0.11, 1.66] mg/mmol; *p*=0.034) across the groups, yet, without significant differences in post hoc tests. Likewise, hsTNT also showed a pattern of gradual increase (median [IQR]: HSP 8.5 [5.8, 9.3] ng/ml; TH 9.0 [8.0, 11.0] ng/ml; MH 9.0 [9.0, 12.0] ng/ml; SL 13.5 [6.0, 16.0] ng/ml; *p*=0.050) and was higher in the SL compared with the healthy cluster (*p*=0.042) (Table 1).

**Fig. 2:**
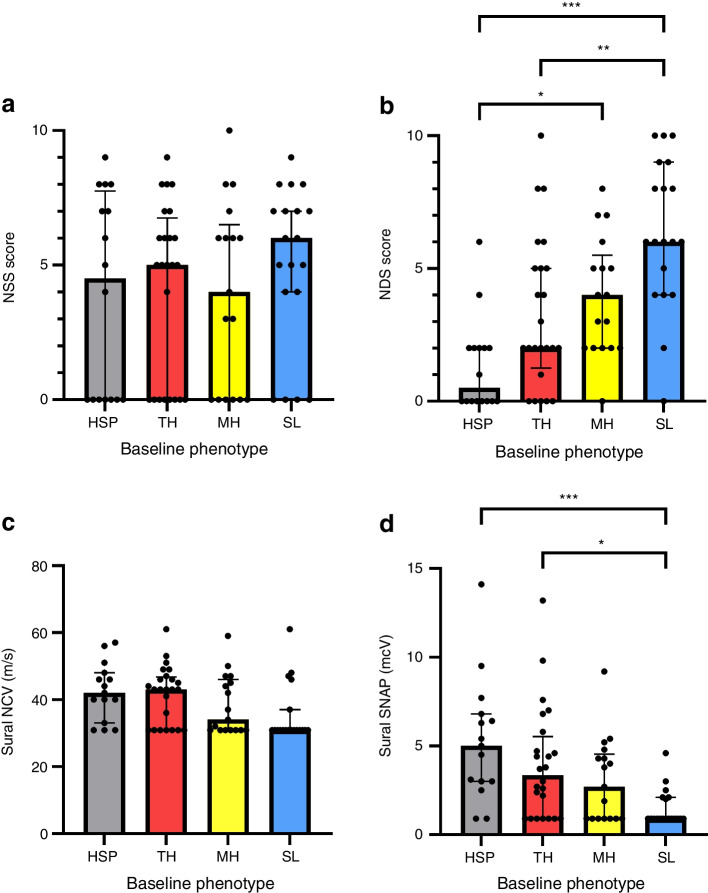
Comparative presentation of clinical neurological and electrophysiological features across the four groups of participants with type 2 diabetes. (**a**) No difference in the severity of neuropathic symptoms across the four phenotypes. (**b**) The magnitude of objective clinical deficits indexed by the NDS shows a gradual increase, culminating in those with SL. (**c**) There are no noted differences in sural NCV. (**d**) Pronounced differences in sural SNAP across the groups, with the greatest magnitude of abnormalities observed in SL. See also Table 1. *p<0.05, **p<0.05, ***p<0.001 in pairwise comparisons after Bonferroni correction

There were no differences across the groups with respect to overall subjective neuropathic symptoms indexed by NSS (Table 1, Fig. 2a). Nevertheless, among NSS-related symptoms, a greater proportion of those with SL reported the presence of pain in lower extremities (Table 1). Objective neurological deficits showed a gradual increase across HSP, TH, MH and SL, being higher in MH compared with HSP and in SL compared with HSP and TH (Table 1, Fig. 2b).

There were no significant differences in sural NCV across the four groups (*p*=0.058), although the prevalence of an abnormally low NCV showed a gradual increase (31.3% vs 33.3% vs 58.8% vs 78.9%, respectively, *p*=0.008), being significantly higher in SL compared with HSP and TH (Table 1, Fig. 2c). In contrast, differences regarding sural SNAP were considerably more pronounced across the groups either as continuous or categorical variables (*p*_ANOVA_=0.001 for both), culminating in lowest and highest values, respectively, in the SL subgroup (Table 1, Fig. 2d). The prevalence of confirmed DSPN according to the Toronto consensus was higher in the SL subgroup compared with all the others, while those in the MH subgroup also showed a higher DSPN frequency compared with those in the HSP subgroup.

**Fig. 3:**
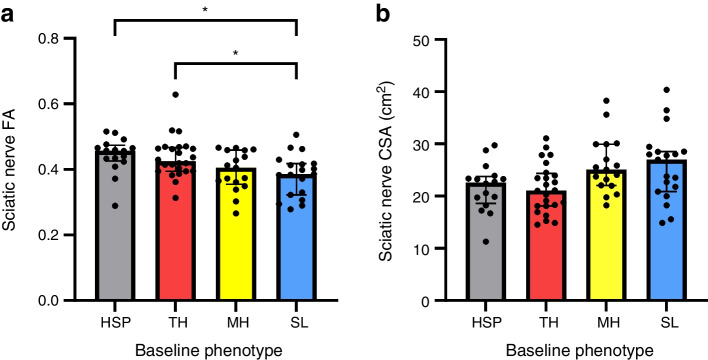
MRN-derived sciatic nerve variables among the different sensory subtypes in participants with type 2 diabetes. (**a**) Disruption of nerve integrity indexed by FA increases across HSP, TH, MH and SL (*p*=0.005), being significantly lower in SL compared with HSP (*p*=0.024) and TH (*p*=0.029). (**b**) An inverse pattern is observed regarding sciatic nerve CSA (*p*=0.012), without significant pairwise differences after Bonferroni correction. *p<0.05 in pairwise comparisons after Bonferroni correction

Analysis of the FA of the sciatic nerve (HSP 0.444, TH 0.437, MH 0.395, SL 0.382; *p*=0.005) revealed a gradual decrease across the clusters (HSP>TH>MH>SL) with a significantly lower FA in SL compared with HSP (*p*=0.024) and TH (*p*=0.029) (Table 1, Fig. 3). In parallel a gradual increase of sciatic nerve CSA at the level of the distal thigh was found (HSP 21.7, TH 21.5, MH 25.9, SL 25.8 mm^2^; *p*=0.011), reaching a maximum in those with MH and SL, yet, without significant differences between subgroups in post hoc testing (Table 1, Fig. 3).

### Comparison with healthy control individuals without type 2 diabetes

Participants with type 2 diabetes were compared with 13 healthy control participants who had similar age, sex and BMI (Table 2). Compared with the sum of the type 2 diabetes groups, there were expected differences in waist circumference, HbA_1c_ and cholesterol levels and the sum of tested peripheral clinical and neurophysiological variables. Accordingly, *z* scores for thermal detection thresholds (cold detection threshold [CDT], warm detection threshold [WDT], thermal sensory limen [TSL]), thermal pain (cold pain threshold [CPT], heat pain threshold [HPT]) and mechanical detection thresholds (mechanical detection threshold [MDT], vibration detection threshold [VDT]), and mechanical pain sensitivity (MPS) were lower among participants with type 2 diabetes, while PHS and DMA were more prevalent among those with type 2 diabetes (ESM Table 1). Additionally, participants with type 2 diabetes exhibited lower FA (0.415 vs 0.459, *p*=0.001) and higher CSA values (23.6 vs 19.9 mm^2^, *p*=0.032) compared with healthy control participants (Table 2).

**Table 2:** Comparisons of demographic, serological, electrophysiological and MRN variables of the sciatic nerve between healthy control participants and the sum of the type 2 diabetes cohort, as well as the HSP group

Variable	Healthy control participants (*n*=13)	T2D-Sum (*n*=76)	T2D-HSP (*n*=16)	*p* value (control participants vs T2D-Sum)	*p* value (control participants vs HSP)
Age, years	59.8 (8.8)	64.7 (8.7)	61.8 (8.7)	0.062	0.907
Female sex, yes, *n* (%)	7 (53.8)	22 (28.9)	5 (31.3)	0.077	0.219
Active smoking, yes, *n* (%)	0 (0.0)	7 (9.2)	1 (6.3)	0.254	0.359
BMI, kg/m^2^	27.8 (5.2)	29.5 (4.3)	29.1 (5.1)	0.240	0.526
Waist, cm	98.7 (15.8)	108.0 (11.8)	103.4 (13.7)	0.015*	0.402
HbA_1c_, mmol/mol	37.6 (3.4)	53.3 (12.5)	53.3 (12.5)	<0.001***	<0.001***
HbA_1c_,%	5.6 (0.3)	7.0 (1.1)	7.0 (0.9)	<0.001***	<0.001***
Τotal cholesterol, mmol/l	5.9 (1.1)	5.0 (1.1)	5.3 (1.3)	0.006**	0.157
eGFR, ml/min per 1.73 m^2^	89.7 (7.9)	88.6 (16.1)	92.5 (12.5)	0.815	0.815
hsCRP, mg/l	0.73 (0.55, 1.38)	1.40 (0.54, 3.47)	1.48 (0.44, 3.54)	0.590	0.590
ACR, mg/mmol	0.09 (0.06, 0.10)	0.11 (0.05, 0.31)	0.07 (0.05, 0.26)	0.210	0.210
hsTNT, ng/ml	6.0 (4.8, 7.0)	9.0 (7.5, 13.0)	8.5 (5.8, 9.3)	0.001**	0.001**
Sural NCV, m/s	46.0 (5.6)	39.5 (8.9)	42.4 (8.6)	0.020	0.191
Sural SNAP, µV	7.3 (2.9)	3.4 (2.9)	5.3 (3.5)	<0.001	0.088
Common peroneal NCV, m/s	43.9 (4.6)	39.5 (6.3)	42.2 (3.7)	0.027	0.304
Common peroneal CMAP, mV	6.6 (2.7)	5.1 (3.1)	7.0 (2.9)	0.150	0.694
Tibial NCV, m/s	45.2 (4.8)	39.4 (5.8)	42.8 (4.1)	0.002**	0.188
Tibial CMAP, mV	18.6 (7.7)	10.4 (6.7)	14.1 (6.7)	<0.001***	0.128
Pain, yes, *n* (%)	0 (0.0)	21 (27.6)	2 (12.5)	0.030*	0.186
ΝSS, *n*/10	0.0 (0.0, 0.0)	5.0 (0.0, 7.0)	4.5 (0.0, 7.8)	0.002**	0.050*
ΝDS, n/10	0.5 (0.0, 1.8)	3.5 (2.0, 6.0)	0.5 (0.0, 2.0)	0.001**	0.698
Pathological sural NCV, yes, *n* (%)	1 (7.7)	37 (48.7)	5 (31.3)	0.005**	0.191
Pathological sural SNAP, yes, *n* (%)	0 (0.0)	38 (50.0)	4 (25.0)	0.001**	0.088
Pathological common peroneal NCV, yes, *n* (%)	0 (0.0)	24 (31.6)	0 (0.0)	0.016*	-
Pathological common peroneal CMAP, yes, *n* (%)	0 (0.0)	9 (11.8)	0 (0.0)	0.184	-
Pathological tibial NCV, yes, *n* (%)	0 (0.0)	30 (39.5)	2 (12.5)	0.005**	0.172
Pathological tibial CMAP, yes, *n* (%)	0 (0.0)	11 (14.5)	1 (6.3)	0.137	0.343
Confirmed neuropathy, yes, *n* (%)	0 (0.0)	30 (39.5)	1 (6.3)	0.005**	0.359
FA	0.459 (0.032)	0.415 (0.065)	0.444 (0.055)	0.001**	0.388
CSA	19.9 (5.0)	23.6 (5.7)	21.7 (4.6)	0.032*	0.331

Values are displayed as *n* (%), means (SD) or medians (25, 75 IQR)

Pathological sural NCV, <38.6 m/s; pathological sural SNAP, <2.54 μV; pathological common peroneal NCV, <37.0 m/s; pathological common peroneal CMAP, <1.14 μV; pathological tibial NCV, <38.0 m/s; pathological tibial CMAP, <2.72 μV

*p<0.05, **p<0.01, ***p<0.001

T2D-HSP, participants with type 2 diabetes and a healthy sensory profile

Group comparison of healthy control individuals with the HSP subgroup yielded higher hsTNT, HbA_1c_ and NSS among the HSP subgroup. QST profiles were also similar between the healthy control and HSP groups, except for slight differences on zTSL (*z* score of TSL; 0.45 vs −0.22, *p*=0.012) and zHPT (*z* score of HPT; 0.88 vs −0.31, *p*=0.036) (ESM Table 1).

### Impact of different sensory phenotypes on FA

Within the cohort of participants with type 2 diabetes, multivariable regression analysis was carried out with sural NCV, sural SNAP and the sciatic nerve’s FA and CSA as the dependent variables and the three sensory phenotypes as independent variables (Table 3). MH and SL were both associated with lower sural SNAP (MH B=−2.2 [95% CI −4.1, −0.3], *p*=0.022; SL B=−3.8 [95% CI −5.6, −1.96], *p*<0.001), lower sciatic FA (MH B=−0.048 [95% CI −0.091, −0.006], *p*=0.027; SL B=−0.062 [95% CI −0.103, −0.020], *p*=0.004) and a higher sciatic CSA (MH B=4.3 [95% CI 0.5, 8.0], *p*=0.028; SL B=4.0 [95% CI 0.4, 7.7], *p*=0.032). Further, SL was associated with a lower sural NCV (B=−7.0 [95% CI −13.0, −1.1], *p*=0.021).

**Table 3:** Multivariable linear regression between sural NCV, SNAP, sciatic FA and sciatic CSA (dependent variables) and the different sensory phenotypes (independent variables; reference group, HSP) among participants with type 2 diabetes

Sensory phenotype	Sural NCV			Sural SNAP			Sciatic FA			Sciatic CSA		
	B	95% CI	*p* value	B	95% CI	*p* value	B	95% CI	*p* value	B	95% CI	*p* value
TH	−0.7	−6.4, 4.9	0.794	−1.3	−3.1, 0.44	0.139	−0.007	−0.046, 0.032	0.724	−0.2	−3.7, 3.3	0.912
MH	−4.0	−10.0, 2.1	0.199	−2.2	−4.1,−0.3	0.022*	−0.048	−0.091, −0.006	0.027*	4.3	0.5, 8.0	0.028*
SL	−7.0	−13.0, −1.1	0.021*	−3.8	−5.6,−1.96	<0.001***	−0.062	−0.103, −0.020	0.004**	4.0	0.4, 7.7	0.032*

*p<0.05, **p<0.01, ***p<0.001

B, unstandardised regression coefficient

After including sural NCV in the multivariable regression model, the association of SL with lower FA remained robust (B=−0.05 [95% CI −0.093, −0.007], *p*=0.023), although that of MH with FA was marginally non-significant (B=−0.042 [95% CI −0.085, 0.001], *p*=0.055) (Table 4). The inclusion of sural SNAP in the multivariable model instead resulted in the abolishment of all associations between sensory phenotypes and FA (Table 4), hence implying that the different phenotypes are distinguished by progressive axonal loss, concurrently reflected by both diminishing SNAP and FA.

**Table 4:** Multivariable linear regression between sciatic FA (dependent variable) and the different sensory phenotypes (reference group, HSP) as well as sural NCV and SNAP (independent variables; reference group, HSP) among participants with type 2 diabetes

Variable	B	95% CI	*p* value
Sural NCV, m/s	0.002	0.000, 0.003	0.040*
TH	−0.006	−0.046, 0.033	0.746
MH	−0.042	−0.085, 0.001	0.055
SL	−0.050	−0.093, −0.007	0.023*
Sural SNAP, μV	0.009	0.004, 0.014	0.001**
TH	0.004	−0.035, 0.042	0.852
MH	−0.030	−0.072, 0.013	0.165
SL	−0.030	−0.074, 0.015	0.185

*p<0.05, **p<0.01, ***p<0.001

B, unstandardised regression coefficient

### Sciatic FA negatively correlates with CSA and markers of microvascular damage

Correlation analysis over the sum of participants with type 2 diabetes revealed that the sciatic FA correlates negatively with the sciatic CSA (*r*=−0.346, *p*=0.002; Table 5), a finding which persisted after adjustment for sex (*r*=−0.269, *p*=0.02). Furthermore, sciatic FA correlated negatively with log_10_(ACR) (*r*=−0.316, *p*=0.006) and the correlation was not substantially affected after adjustment for HbA_1c_ and log(type 2 diabetes duration) (*r*=−0.328, *p*=0.009). FA also showed a negative correlation with log_10_(hsTNT) (*r*=−0.382, *p*=0.002) and the correlation remained robust after adjusting for age and eGFR (*r*=−0.317, *p*=0.014).

**Table 5:** Correlations of the sciatic nerve’s FA of all study participants with type 2 diabetes with demographic, instrumental-based, clinical and serological variable

Variable	FA	Age	Female sex	log_10_(diabetes duration)	BMI	Waist	Cholesterol	HbA_1c_	eGFR	log_10_(ACR)	log_10_(hsTnT)
CSA	−0.346**	0.025	0.81**	0.077	0.072	0.232*	−0.149	0.015	0.181	0.119	0.195
FA		−0.143	1.01*	0.153	−0.030	−0.162	0.034	−0.073	0.032	−0.316**	−0.382***
Age			0.97	0.093	−0.284*	−0.052	−0.117	0.099	−0.592***	0.144	0.461***
Female sex				3.90	1.07	0.97	1.00	1.43	0.98	0.68	0.06
log_10_(diabetes duration)					0.246*	0.207	0.055	0.282*	0.015	0.040	−0.028
BMI						0.823***	0.150	0.110	0.127	0.128	−0.135
Waist							0.038	0.071	0.114	0.151	0.094
Chole								0.134	0.011	0.019	−0.064
HbA_1c_									0.026	0.310***	0.105
eGFR										0.017	−0.320*
logACR											0.126
logTnT											

Values are Pearson’s *r* for correlations between continuous variables or OR for categorical variables (sex)

*p<0.05, **p<0.01, ***p<0.001

## Discussion

This study is the first to investigate associations of sensory phenotyping through QST supplemented by DTI-MRN at 3T. The main findings were as follows: (1) in individuals with type 2 diabetes the sciatic nerve’s FA gradually decreases across the sensory phenotypes (HSP>TH>MH>SL) in conjunction with increasing clinical deficits and increased DSPN prevalence before the onset of subjective symptoms; (2) MH and SL are both associated with lower sural SNAP, lower sciatic FA and a higher sciatic CSA, all of which may serve as an additional marker of structural deterioration, while SL was also associated with a lower sural NCV; (3) over all participants the sciatic nerve’s FA negatively correlates with sciatic CSA and markers of microvascular damage, namely ACR and hsTNT. Furthermore, electrophysiological analyses suggest that diminishing FA across the different sensory phenotypes is mediated by progressive axonal loss and constitutes the structural and functional equivalents of sensory nerve damage in DSPN.

Although comparison with a cohort of control participants with similar age, sex and BMI revealed higher sciatic nerve FA and lower CSA compared with the sum of participants with type 2 diabetes, indicating greater structural integrity, there was no difference in sciatic FA and CSA compared with HSP (Table 2). This further indicates that the peripheral sensory profile is decisively determined by the degree of structural nerve damage, rather than by type 2 diabetes diagnosis or other potential confounders accompanying the type 2 diabetes status.

The finding that the FA of the sciatic nerve as a measure of peripheral nerve integrity [14, 15] was lower in SL compared with HSP and TH in combination with MH and SL being associated with lower sciatic FA values in multiple regression analysis suggests that individuals with type 2 diabetes may experience a predefined continuous cascade of nerve fibre damage in the course of the disease from HSP to TH, to MH and finally SL. Previous studies showed that predominant small-fibre damage is an early finding in individuals with diabetes [23, 24], and may be observed in those who primarily suffer from TH, which is mediated by an impairment of thin myelinated Aδ fibres and small unmyelinated C fibres. Hence, the fact that FA was not lower in the TH group compared with HSP group may potentially be explained through a low cumulative amount of nerve fibre damage with retained nerve structural integrity in this early disease stage. This is underlined by the result that TH was also not associated with lower sural NCV and SNAP, in line with the assumption that the cumulative nerve fibre damage is relatively sparse and small-fibre-dominant and can therefore not be detected by nerve conduction studies [25, 26]. Nonetheless, in MH, which is defined by loss of small-fibre function and mechanical allodynia/hyperalgesia, and in SL, which is characterised through small- and large-fibre loss [7, 18], FA was lower than in the HSP group, suggesting that a progression of small-fibre damage and involvement of large-fibres occur in the transition from MH to SL resulting in a lower structural nerve integrity of the sciatic nerve. Furthermore, sural SNAP was found to considerably differ across the four phenotypes, with a gradual monotonous decrease across HSP, TH, MH and SL, while differences regarding NCV were far more subtle. Likewise, the significant associations of MH and SL with a lower FA were largely retained after adjustment for sural NCV. However, this was not the case for sural SNAP, the inclusion of which in the multivariable model attenuated the aforementioned findings. SNAP reductions constitute an earlier feature of axonal loss in DSPN, temporally preceding the decrease in NCV [27]. Taken together, this evidence further adds to the notion that the patterns of diminishing FA across the different sensory phenotypes are mediated by progressive axonal loss and constitute the structural and functional equivalents of sensory nerve damage in DSPN. This is supported by a previous study which found that the amount of sciatic nerve lesions diagnosed through high-resolution MRN is associated with the severity of DSPN indicating that peripheral nerve damage may accumulate in the course of the disease and determine the onset and severity of DSPN [11]. In addition, a gradual increase of the NDS, with significantly higher scores in participants with MH and SL compared with HSP, was found and further indicates a higher degree neurological deficit in individuals with MH and SL. In further support of this observation, the prevalence of confirmed DSPN was highest in those with SL and higher in those with MH than in the HSP subgroup (Table 1).

Our hypothesis finds further support in the finding that a higher sciatic CSA is associated with sensory profiles of increasing neuropathic severity such as MH and SL and that the sciatic CSA correlates inversely with sciatic FA in a correlation analysis over all participants with type 2 diabetes. Several studies on peripheral nerve pathologies of various aetiologies previously demonstrated that an increase of the CSA was associated with more severe nerve damage [28–32]. A greater peripheral (most prominently, sciatic or tibial) nerve CSA assessed by ultrasound or MRN has been also ascertained in DSPN compared with control individuals with HSP, albeit with considerable overlap of CSA values and without convincing evidence of an association between CSA and the severity of DSPN [33–35]. Moreover, in individuals with carpal tunnel syndrome, an inverse correlation of the nerve’s FA and CSA was found, with the FA being associated with a higher severity of neurological deficits [36]. However, our study is the first to show such correlation in individuals with polyneuropathy, underlining that an increased CSA is associated with a compromised nerve function in individuals with polyneuropathies such as DSPN. Subsequently it may be hypothesised that the CSA of peripheral nerves gradually increases in the course of type 2 diabetes while the structural integrity gradually decreases.

We ascertained a negative correlation between the sciatic FA and markers of microvascular damage, namely hsTNT and ACR. Furthermore, perturbations of those markers were found to gradually increase in severity across HSP, TH and MH and peak in those with SL, thus exhibiting a pattern similar to FA and SNAP. This may indicate that microangiopathic mechanisms may play a primary role in nerve damage and sensory dysfunction in type 2 diabetes, as suggested by previous studies using MRN [10, 12, 37, 38]. Even though no association between HbA_1c_ and FA or differences among sensory phenotypes were found in the current study, the positive correlation between HbA_1c_ and ACR highlights the implication of hyperglycaemia in the pathogenesis of microangiopathy.

Our results add to the findings of previous studies and emphasises the advantages of disease stratification through sensory phenotypes, potentially allowing a more individualised severity-adapted management of individuals with type 2 diabetes and DSPN [7, 39, 40].

Strengths of our study include the implementation of comprehensive measures of nerve structural and functional assessment (MRN, nerve conduction studies, QST) for a detailed phenotyping of participants in our cohort. Further, comparative analysis was conducted among participant subgroups exhibiting different sensory phenotypes but they did not differ regarding several variables pertinent to nerve damage in type 2 diabetes such as age, sex, the number of active smokers per group, BMI, waist circumference, duration of type 2 diabetes, HbA_1c_, eGFR and hsCRP. This allowed for unconfounded observations regarding our study hypothesis, despite the relatively small sample size.

Some limitations have to be acknowledged. First, due to the cross-sectional design of the study we cannot draw definite conclusions concerning a distinct cascade of nerve damage and sensory profiles through which individuals with type 2 diabetes pass until they experience a complete loss of sensory nerve fibre function. However, the increase in the severity of objective findings from TH over MH towards SL suggests a natural course of DSPN involving TH and MH as transitional phenotypes. Additionally, not all potential confounding factors in this study can be precluded due to the sample size. However, compared with other studies, participants were well phenotyped according to established factors that exert an impact on the natural course of DSPN. Furthermore, we lack data from subgroups with younger age or lower BMI, both of which would have contributed to the generalisability of our results. Nonetheless, the compared groups did not differ regarding age and BMI, while no correlations were found between those factors and FA or CSA and hence it is unlikely that this limitation constitutes a significant source of bias for the current study. Another limitation of the study is the lack of implementation of specialised scales or questionnaires to assess the presence and intensity of pain in lower extremities, other than reported pain as part of the NSS score. QST is limited by dependence on an individual’s attentiveness and compliance, as well as variable reproducibility. Nonetheless, QST was carried out by experienced and trained personnel and was strictly adherent to the protocol of DFNS, in order to minimise reproducibility issues. Further, QST represents a highly sophisticated method. Nevertheless, it is also a notably labour-intensive approach demanding personnel with specialised training. As a result, the application of QST is reserved for settings with specific clinical indications in specialised centres as well as clinical research. Another important aspect is that the sample size of the study cohort did not allow us to conduct analyses separated for sex or gender. Subsequently, our results do not provide insights into gender- or sex-specific differences regarding associations of sensory phenotypes with the structural integrity of peripheral nerves.

In summary, our study represents the first to find associations of the structural integrity of peripheral nerves with sensory profiles based on QST as suggested by Baron et al [7] indicating that individuals with type 2 diabetes may pass through a predefined cascade of nerve damage from HSP, to symptoms of predominating TH to predominating MH and ultimately experience complete loss of sensory sensation. These patterns of sensory clusters may be accompanied by a gradual decrease of structural integrity of the sciatic nerve as measured by the FA, mediated by progressive axonal loss, along with a simultaneous increase of the nerve’s CSA. However, longitudinal data are required to confirm whether nerve damage follows a certain cascade predominantly mediated by nerve fibre loss.

### Supplementary Information

Below is the link to the electronic supplementary material.

Supplementary file1 (PDF 31 KB)

## Data Availability

Data will be provided by the corresponding author upon reasonable request.

## Abbreviations

ACR: Albumin/creatinine ratio
CMAP: Compound motor action potential
CDT: Cold detection threshold
CPT: Cold pain threshold
CSA: Cross-sectional area
DFNS: German Research Network on Neuropathic Pain
DMA: Dynamic mechanical allodynia
DSPN: Diabetic sensorimotor polyneuropathy
DTI: Diffusion tensor imaging
FA: Fractional anisotropy
HEIST-DiC: Heidelberg Study on Diabetes and Complications
HPT: Heat pain threshold
hsCRP: High-sensitivity C-reactive protein
HSP: Healthy sensory profile
hsTNT: High-sensitivity troponin T
MDT: Mechanical detection threshold
MH: Mechanical hyperalgesia
MPS: Mechanical pain sensitivity
MRN: Magnetic resonance neurography
NCV: Nerve conduction velocity
NDS: Neuropathy disability score
NSS: Neuropathy symptom score
PHS: Paradoxical heat sensations
QST: Quantitative sensory testing
SL: Sensory loss
SNAP: Sensory nerve action potential
T2w: T2-weighted
TH: Thermal hyperalgesia
TSL: Thermal sensory limen
VDT: Vibration detection threshold
WDT: Warm detection threshold
WUR: Wind-up ratio
