# Supplementary material for: A diminished sciatic nerve structural integrity is associated with distinct peripheral sensory phenotypes in individuals with type 2 diabetes
Source: Diabetologia. 2023 Nov 29;67(2):275–89. doi: 10.1007/s00125-023-06050-y (PMC10789832; doi:10.1007/s00125-023-06050-y)
Supplement: Supplementary file 1 — Supplementary file1 (PDF 31 KB) [file 125_2023_6050_MOESM1_ESM.pdf]

Electronic supplementary material

ESM Results

QST profiles of each subgroup among T2D participants are presented. Apart from MPT and WUR, significant differences were noted across the four subgroups in all other QST domains. All z-scores of thermal detection thresholds (CDT, WDT, TSL) were significantly lower in MH and SL compared to HSP, while WDT was also lower in TH than healthy. Unsurprisingly, z-scores for thermal pain (CPT, HPT) were significantly higher in those with TH compared with the other two neuropathic phenotypes, signifying hypersensitivity towards thermal pain stimuli. Mechanical detection thresholds (MDT, VDT) were lower in MH than healthy and lowest in SL compared with all other subgroups. Regarding mechanical pain domains, MPT was lower in SL than TH while MPS were significantly lower in SL compared with all other subgroups.

ESM Tables

	Control subjects	T2DM-Sum	T2DM-HSP	T2DM-TH	T2DM-MH	T2DM-SL	P-ANOVA%	P-Healthy controls vs. T2DM-Sum	P healthy controls vs. T2DM-HSP
zCDT	0.23 (0.90)	-1.10 (1.32)	0.13 (0.82)	-0.51 (0.86)	-1.74 (1.02)***##	-2.31 (1.04)***###	<0.001***	0.001**	0.752
zWDT	0.19 (1.20)	-1.07 (0.79)	-0.23 (0.67)	-0.95 (0.62)**	-1.30 (0.65)***	-1.72 (0.47) ***###	<0.001***	<0.001***	0.239
zTSL	0.45 (0.71)	-1.02 (0.93)	-0.22 (0.62)	-0.68 (0.66)	-1.35 (0.74)***	-1.81 (0.86)***###	<0.001***	<0.001***	0.012*
zCPT	0.25 (1.13)	-0.35 (0.91)	-0.29 (0.82)	0.25 (1.09)	-0.56 (0.67)***#	-0.97 (0.16) ###	<0.001***	0.035*	0.151
zHPT	0.88 (1.62)	-0.67 (1.22)	-0.31 (1.24)	-0.07 (1.55)	-1.12 (0.62)#	-1.30 (0.57)###	0.001**	<0.001***	0.036*
zPPT	-0.21 (1.51)	-0.58 (1.17)	-0.53 (1.39)	-0.13 (1.14)	-0.50 (0.88)#	-1.25 (0.97)##	0.016*	0.319	0.559
zMPT	1.14 (1.38)	1.55 (2.10)	2.03 (1.78)	1.55 (2.14)	1.55 (2.10)	0.17 (2.73)##	0.076	0.801	0.151
zMPS	1.00 (1.22)	0.77 (1.63)	1.68 (1.62)	1.01 (1.45)	1.26 (0.95)	-0.72 (1.41)***##$$$	<0.001***	0.631	0.224
zWUR	0.17 (1.14)	0.16 (1.07)	0.30 (1.46)	0.09 (0.69)	0.26 (1.03)	0.02 (1.23)	0.866	0.984	0.791
zMDT	0.33 (0.90)	-1.14 (1.50)	0.08 (1.04)	-0.83 (1.21)	-1.23 (0.78)*	-2.45 (1.65)*** ###$	<0.001***	0.001**	0.496
zVDT	-0.20 (1.35)	-1.97 (2.40)	-0.16 (1.55)	-1.69 (1.91)	-2.18 (2.57)*	-3.67 (2.29)***#	<0.001***	0.011*	0.947
zPHS	0.15 (0.55)	1.00 (1.17)	0.75 (1.12)	0.88 (1.08)	1.29 (1.36)	1.11 (1.15)	0.531	0.009**	0.165
zDMA	0.00 (0.00)	1.88 (5.70)	1.48 (3.45)	2.63 (7.36)	2.14 (7.53)	1.09 (2.31)	0.835	0.005**	0.107

ESM Table 1:

Values of the 13 QST domains compiling the QST profiles of healthy controls, the sum of T2D cohort (T2DM-Sum) and subgroups of T2D participants per sensory phenotype.

T2DM-HSP: individuals with type 2 diabetes and healthy sensory profile, T2DM-TH: individuals with type 2 diabetes and thermal hyperalgesia, T2DM-MH: individuals with type 2 diabetes and mechanical hyperalgesia, T2DM-SL: individuals with type 2 diabetes and sensory loss. zCDT: z-score of cold detection threshold, zCPT: z-score of cold pain threshold, zDMA: z-score of dynamic mechanical allodynia, zMDT: z-score of mechanical detection threshold, zMPS:

z-score of mechanical pain sensitivity, zMPT: s-score of mechanical pain threshold, zPHS: z-score of paradoxical heat sensations, zPPT: z-score of pain threshold, zVDT: z-score of vibration detection threshold, zWDT: z-score of warm detection threshold, zWUR: z-score of wind-up ratio.

[%]P-values obtained from a comparison across the four phenotypes of T2D (T2D-HSP, TH, MH and SL) using once-way analysis of variance

*, **, ***: p values below 0.05, 0.01, 0.001, respectively, for difference vs. the T2D-HSP subgroup in post-hoc pairwise comparisons after Bonferroni correction

#, ##, ##: p values below 0.05, 0.01, 0.001, respectively, for difference vs. the TH subgroup in post-hoc pairwise comparisons after Bonferroni correction

$, $$, $$$: p values below 0.05, 0.01, 0.001, respectively, for difference vs. the MH subgroup in post-hoc pairwise comparisons after Bonferroni correction
